# DNA Methylation Affects the Efficiency of Transcription Activator-Like Effector Nucleases-Mediated Genome Editing in Rice

**DOI:** 10.3389/fpls.2017.00302

**Published:** 2017-03-13

**Authors:** Hidetaka Kaya, Hisataka Numa, Ayako Nishizawa-Yokoi, Seiichi Toki, Yoshiki Habu

**Affiliations:** ^1^Institute of Agrobiological Sciences, National Agriculture and Food Research Organization (NARO)Tsukuba, Japan; ^2^Advanced Analysis Center, National Agriculture and Food Research Organization (NARO)Tsukuba, Japan

**Keywords:** DNA methylation, genome editing, methylcytosine-recognition module, rice, TALENs

## Abstract

Genome editing in plants becomes popular since the advent of sequence-specific nucleases (SSNs) that are simple to set up and efficient in various plant species. Although transcription activator-like effector nucleases (TALENs) are one of the most prevalent SSNs and have a potential to provide higher target specificity by their dimeric property, TALENs are sensitive to methylated cytosines that are present not only in transposons but also in active genes in plants. In mammalian cells, the methylation sensitivity of TALENs could be overcome by using a base-recognition module (N^∗^) that has a higher affinity to methylated cytosine. In contrast to mammals, plants carry DNA methylation at all cytosine contexts (CG, CHG, and CHH, where H represents A, C, or T) with various degrees and effectiveness of N^∗^ module in genome editing in plants has not been explored. In this study, we designed sets of TALENs with or without N^∗^ modules and examined their efficiency in genome editing of methylated regions in rice. Although improvement in genome editing efficiency was observed with N^∗^-TALENs designed to a stably methylated target, another target carrying cytosines with various levels of methylation showed resistance to both normal and N^∗^-TALENs. The results suggest that variability of cytosine methylation in target regions is an additional factor affecting the genome editing efficiency of TALENs.

## Introduction

Genome editing in crops requires the introduction of sequence-specific nucleases (SSNs) into plants via transformation, commonly involving *Agrobacterium*-mediated systems. Rice and other crops are transformed via callus—a mass of dedifferentiated cells from which a whole plant body can be regenerated. Recently, methylation profiles in the genome of rice callus have been reported, showing that methylation state in a fraction of the genome is unstable whereas methylation in the other majority of the genome is highly reproducible in independent calli (Stroud et al., 2013). This provides a great opportunity to examine the effects of target site methylation status on genome editing in rice.

The genome editing systems currently most widely used in plants are clustered regularly interspaced short palindromic repeat (CRISPR)/CRISPR-associated protein (Cas) and transcription activator-like effector nucleases (TALENs; Boettcher and McManus, 2015). TALENs utilize a combination of 7–34 nucleotide recognition modules and have potential to provide higher specificities than CRISPR/Cas. The lower constraint in selecting target sites for TALENs, and the requirement for dimerization of the *Fok*I nuclease portion of TALENs further suppresses off-target digestion of monomer binding sites (Moscou and Bogdanove, 2009; Boettcher and McManus, 2015). However, one disadvantage of TALENs is the low affinity of their cytosine-recognition module for methylated cytosine (Valton et al., 2012), which is commonly found in the genomes of plants and vertebrates (Law and Jacobsen, 2010). Binding of CRISPR/Cas to target regions in the genome is also influenced by chromatin environment (Kuscu et al., 2014), suggesting that epigenome state plays a role in efficient genome editing.

## DNA Methylation Affects the Efficiency of Genome Editing with Talens

**FIGURE 1:**
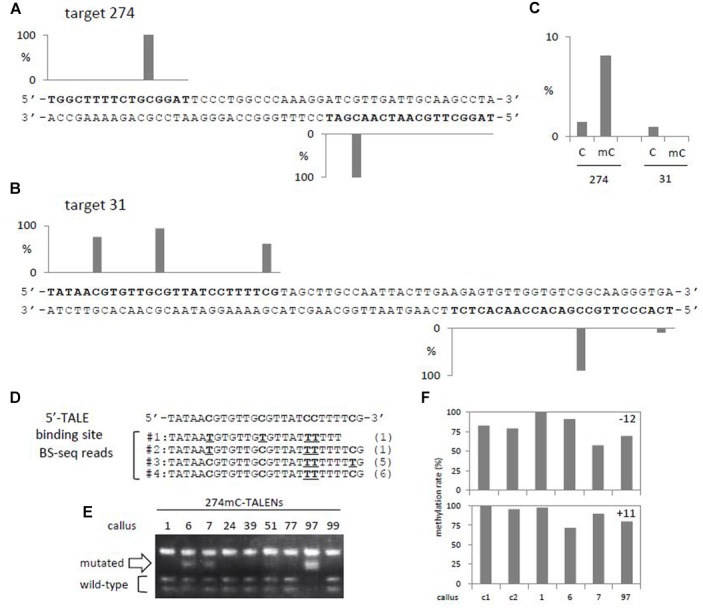
**Methylation status of target regions and efficiency of genome editing by TALENs. (A,B)** Methylation rates of cytosines in 5′- and 3′-TALE binding sites (bold) of target 274 **(A)** and 31 **(B)** regions were shown in graphs. Methylation rates obtained from two BS-seq data of wild-type rice callus samples are combined (GSM1039499 and GSM1039500 by Stroud et al., 2013). **(C)** Efficiency of genome editing by TALENs containing cytosine- (denoted as C) or methylcytosine-modules (denoted as mC) designed for targets 274 and 31 (Supplementary Table S3). **(D)** Sequences of BS-seq reads of rice callus mapped to 5′-TALE binding region in target 31. Nucleotides representing methylated cytosines (denoted as C) and unmethylated/bisulfite-converted cytosines (denoted as underlined T) in BS-seq reads are shown bold. Numbers of reads carrying corresponding sequences are shown in parenthesis. **(E)** CAPS analysis of target 274 region of calli carrying 274mC-TALENs. Callus clone 97 shows the highest efficiency of sequence changes at the target site and clones 6 and 7 show relatively low frequencies (Supplementary Table S4). **(F)** Changes in methylation rates of cytosines around target 274. Methylation rates of highly methylated cytosines at CG context in target 274 **(A)** were analyzed by bisulfite sequencing, and results of calli carrying a control TALENs (calli c1 and c2) or 274mC-TALENs (calli No. 1, 6, 7, and 97) are shown (Supplementary Tables S4–S6). Upper panel, position -12 in the 5′-TALE binding region; lower panel, position +11 in the 3′-TALE binding region **(A)**.

To examine the effect of DNA methylation on the efficiency of TALEN-mediated genome editing in rice, we selected a gene encoding acetyl-CoA synthetase1 (*ACS1*, *Os02g0525900/LOC_Os02g32490*), which is highly methylated at CG sites within its gene body, a character typical of active genes (Supplementary Figure S1; Feng et al., 2010; Zemach et al., 2010). We selected two methylated regions within the gene body of *ACS1* as targets that contain at least one highly methylated cytosine in each TALE-binding region (**Figures 1A,B**). Examination of two target sites located at close proximity within a gene is expected to minimize differences due to factors other than cytosine methylation, such as higher order chromatin structure and transcription state, and to provide an accurate estimation of the effect of cytosine methylation on the efficiency of genome editing by TALENs. Plasmids carrying N^∗^ modules of TALENs were created by deleting a codon corresponding to glycine at the 13th position in NG modules (Valton et al., 2012). TALE binding domains were constructed by the Golden Gate system, and fused with heterodimer-type *Fok*I (Cermak et al., 2011; Zhang et al., 2013). Calli carrying TALENs designed to target a region in the *waxy* gene (L1-R2; Nishizawa-Yokoi et al., 2016) were used as controls. *Agrobacterium*-mediated transformation was performed as described (Toki et al., 2006).

One of the target regions of TALENs (target 274) contains two CG sites. BS-seq data of two independent wild-type callus samples showed that both cytosines are highly methylated in callus (**Figure 1A**; Stroud et al., 2013). The second target region (target 31) contains four CG sequences (three in the left and one in the right target sequence), and all are methylated to various levels (**Figure 1B**). In addition, the target 31 region contains an additional CHG site that is methylated at 10% in callus. No other cytosines in the target regions are methylated. Stability of the methylation states of these target regions were confirmed by another two independent BS-seq data of wild-type callus samples; methylation in target 274 is relatively stable whereas that of target 31 is variable (Supplementary Figure S2).

In TALEN-mediated genome editing in mammalian cells, inefficient binding of TALENs to targets containing methylated cytosines could be improved by using an N^∗^ module that lacks an amino acid residue within the repeated variable diresidue functioning in base recognition (Valton et al., 2012). We prepared a set of TALENs for each target: one contains HD modules that bind to non-methylated cytosine, and the other contains N^∗^ modules at the positions corresponding to cytosines in the methylated CG sites (Supplementary Table S1). Although the N^∗^ module also binds to thymine (Moscou and Bogdanove, 2009), replacement of HD modules with N^∗^ modules did not lead to drastic changes in potential off-target sites in the rice genome (Supplementary Table S2). T-DNAs encoding these TALENs were introduced into rice calli and accumulation of the TALENs mRNA was confirmed by RT-PCR (Supplementary Figure S3).

For target 274, efficient genome editing was observed with N^∗^ module-containing TALENs (274mC-TALENs) but not HD module-containing TALENs (274C-TALENs) (**Figure 1C**, Supplementary Figure S4 and Table S3). Alterations in the target 274 sequence by 274mC-TALENs were confirmed by cloning and sequencing of amplified fragments from calli (Supplementary Figures S4B–D). Conversely, for target 31, neither HD module-containing TALENs (31C-TALENs), nor N^∗^ module-containing TALENs (31mC-TALEN) yielded detectable genome editing (**Figure 1C**, Supplementary Figure S5 and Table S3). Since target 31 contains five cytosines that are methylated at various levels in callus (**Figure 1B**), we analyzed a combination of unmethylated and methylated cytosines in each single DNA molecule by aligning reads of BS-seq that cover the 5′-TALE binding region of target 31. As expected, the three cytosines in CG context are differentially methylated in various combinations in each read (**Figure 1D**). The number of reads carrying methylated cytosines at all three CG sites was less than half of the total reads covering the 5′-TALE binding region. The second most abundant read contained two methylated cytosines and one unmethylated cytosine at CG sites, indicating that the corresponding DNA molecule in the genome carries mixtures of methylated and unmethylated cytosines in the TALE-binding region and so would not be an optimal target for either 31C- or 31mC-TALENs. The 3′-TALE binding region of target 31 contains two cytosines methylated at 90 and 10%, respectively (**Figure 1B**), and this would add further variations to combinatorial binding sites for 31C- and 31mC-TALENs. Together, these data indicate that the presence of methylated cytosines at various levels affects the efficiency of genome editing with TALENs.

To examine the observed inverse correlation between methylation levels in target regions and efficiency in TALEN-medicated genome editing further, we surveyed published studies in which efficiencies of TALENs without the N^∗^ module were examined in rice (Nishizawa-Yokoi et al., 2016; Zhang et al., 2016), and indeed found an inverse correlation between methylation rate in target regions and efficiency in TALEN-mediated genome editing (Supplementary Figures S6, S7). The data are consistent with a previous observation in mammalian cells (Valton et al., 2012) and support our notion that cytosine methylation could impede efficiency of TALEN-mediated genome editing in rice.

## Genome Editing Changes the Methylation State in Close Proximity to Target Site

Double-stranded breaks (DSBs) introduced by SSNs are repaired by homologous recombination (HR) or non-homologous end joining (NHEJ); HR restores original sequences, whereas NHEJ often results in various lengths of deletions/insertions. Introduction of mutations by TALENs implies that NHEJ is, at least partly, involved in repair of DSBs by TALENs. Resection of DSB occurs in both repair pathways and is followed by synthesis of new DNA strands in HR; a process that would erase epigenetic information around DSB sites. In addition, DSB formation recruits a set of chromatin proteins that transiently modifies the surrounding chromatin state in eukaryotic cells (Ayrapetov et al., 2014). Therefore, we examined whether the methylation state of cytosines around the target site of 274mC-TALENs changes upon TALEN-mediated genome editing in rice. Bisulfite sequencing analysis of independent calli with various levels of mutation frequencies (**Figure 1E**) showed that methylation rate of cytosine in the 5′ binding site of target 274 (position -12) is high but slightly variable since even control calli showed reduced levels of methylation (c1 and c2 in **Figure 1F**, upper) that is consistent with data obtained in four independent BS-seq data (Supplementary Figure S2). In contrast, a cytosine in the 3′ binding site of target 274 (position +11) was stably maintained in control calli and a transformed callus carrying no detectable mutation (callus No. 1; **Figures 1E,F**, lower). Among these calli, a callus sample carrying an active 274mC-TALENs (callus No. 6; **Figure 1E**) showed statistically significant differences in methylation at position +11 relative to those of controls and callus without detectable mutation (Supplementary Tables S4,S5). Although, the slight variation even in the control calli made the result obscure, the results indicate that genome editing by TALENs in rice might change the epigenetic state in close proximity to target sites (at least position +11) in rice as reported in mammalian cells (Ayrapetov et al., 2014).

## Prospect

Plants have methylated cytosines at all sequence contexts (CG, CHG, and CHH) and gene body CG methylation is frequently observed in active genes (Feng et al., 2010; Zemach et al., 2010). Although the role of gene body methylation is currently unknown, methylated cytosines are expected to be bound by methylcytosine-binding proteins, resulting in chromatin that is less accessible for SSNs *in vivo*. Our data provide a precedent for plant genome editing by TALENs in which the methylation state is a critical determinant in selecting target regions and choice of cytosine- or methylcytosine-binding modules in TALENs. A combination of TALENs and information on methylation state in callus will lead to improved efficiencies for genome editing in crops.

## Author Contributions

YH designed the work; HK, AN-Y, and YH conducted the experiments; HN analyzed the data; HK, HN, AN-Y, ST, and YH drafted, reviewed, and edited the manuscript.

## Conflict of Interest Statement

The authors declare that the research was conducted in the absence of any commercial or financial relationships that could be construed as a potential conflict of interest.
