# Supplementary material for: DNA Methylation Affects the Efficiency of Transcription Activator-Like Effector Nucleases-Mediated Genome Editing in Rice
Source: Front Plant Sci. 2017 Mar 13;8:302. doi: 10.3389/fpls.2017.00302 (PMC5346537; doi:10.3389/fpls.2017.00302)
Supplement: Supplementary file 1 [file Presentation_1.pdf]

Supplementary Material

DNA methylation affects the efficiency of transcription activator-like effector nucleases-mediated genome editing in rice

Hidetaka Kaya, Hisataka Numa, Ayako Nishizawa-Yokoi, Seiichi Toki, Yoshiki Habu*

*** Correspondence:** Yoshiki Habu: email habu@affrc.go.jp

1 Supplementary Tables and Figures

1.1 Supplementary Tables

Supplementary Table S1. Sequences of TALE-modules in TALENs used in this study.

Supplementary Table S2. Off-target analysis of TALENs used in this study.

Supplementary Table S3. Frequency of TALENs-induced mutations in *ACS1*.

Supplementary Table S4. Summary of mutation frequencies and CG methylation rates around target 274 in calli carrying a control TALENs and 274mC-TALENs.

Supplementary Table S5. Effect of genome editing on methylation of cytosine at +11 position in target 274.

Supplementary Table S6. Primers used in this study.

1.2 Supplementary Figure

Supplementary Figure S1. Methylation state of *ACS1* gene region.

Methylation rates (red bars) of cytosines in CpG, CHG, and CHH contexts in *ACS1* gene region are separately shown. Exon-intron structures annotated in RAP and MSU databases are shown. OT, top strand; OB, bottom strand. BS-seq data of rice callus were taken from GSM1039499 (Stroud et al, 2013).

Supplementary Figure S2. Methylation rates of individual cytosines in the target regions in *ACS1*.

Methylation rates of cytosines in the target regions of TALENs used in this study were extracted from four independent BS-seq data of wild-type rice calli (blue bars, Stroud et al 2013; green bars, BS-seq that was done in this study with two independent wild-type Nipponbare callus samples (4 weeks after callus induction) as described previously (Numa et al 2015). Positions of cytosines in the TALE-binding regions are shown with the context of each cytosine (CG, CHG, or CHH). (**A**) target 274; (**B**) target 31.

Supplementary Figure S3. RT-PCR of introduced TALENs constructs.

RT-PCR was performed for *TALENs* (upper), *NPTII* (middle), and endogenous *ACTIN* (bottom) genes. (**A**) calli transformed with TALENs constructs for target 274; (**B)** calli transformed with TALENs constructs for target 31.

Supplementary Figure S4. Mutations induced by 274mC-TALENs.

(**A**) CAPS analysis of target 274 region in calli carrying 274C- or 274mC-TALENs. Changes in nucleotide sequences at the target sequence are expected to produce DNA fragments around 290 bp. Upper, 274C-TALENs containing cytosine-binding modules; lower, 274mC-TALENs containing methylcytosine-binding modules; m, a 100 bp-size marker. Results of representative 24 colonies for each TALENs are shown. Numbers of calli showing sequence alterations at the target site are indicated in red. (**B**) Sequences of deletions induced by 274mC-TALENs. Numbers of deleted nucleotides (del) and numbers of PCR clones carrying corresponding sequences (n) are indicated to the right. Left and right binding sites of 274mC-TALENs are shown in red. A *Hae*III site (GGCC)

located at the center of left- and right-binding sites was used for the CAPS analysis. (**C**) Sequences of insertions induced by 274mC-TALENs. Numbers of inserted nucleotides (in) are indicated to the right. Inserted nucleotides are indicated in blue. (**D**) Sequence rearrangements induced by 274mC-TALENs. Nucleotides inserted into the target site are indicated in brown or green for each clone. Positions of sequences that are homologous to the inserted sequences are indicated in same colors. Sizes of insertions/deletions (in/del) are shown in the right.

Supplementary Figure S5. CAPS analysis of target 31 region.

Changes in nucleotide sequences at the target sequence are expected to produce DNA fragments around 470 bp. (**A**) 31C-TALENs containing cytosine-binding modules; (**B**) 31mC-TALENs containing methylcytosine-binding modules; m, a 100 bp-size marker. Results of representative 24 colonies for each TALENs are shown.

Supplementary Figure S6. Inverse correlation between mutation frequencies in TALEN-mediated genome editing and methylation rates of cytosines in the target regions in rice.

(upper) Mutation frequencies of TALEN-mediated genome editing taken from a published study. L1-R2 and L2-R2, Nishizawa-Yokoi et al (2016); others, Zhang et al (2016). (lower) Methylation rates of cytosines in the target regions. Methylation rates of the target regions in wild-type rice calli were calculated by combining four independent BS-seq data: two from Stroud et al (2013) and two obtained in this study. Names of TALENs are shown at the bottom with their target genes/regions and nucleotide sequences of the target regions are shown in Supplementary Figure S7. Those shown in red are TALENs with detectable genome editing activities. L1-R2 has a higher genome editing activity, indicating that there would be factors other than cytosine methylation that affect efficiency of TALENs.

Supplementary Figure S7. Nucleotide sequences of target regions of TALEN-mediated genome editing.

(**A**) Zhang et al (2016) and (**B**) Nishizawa-Yokoi et al (2016). Nucleotides in left and right TALEN-binding regions are shown in bold letters.

Supplementary Table S1. Sequences of TALE-modules in TALENs used in this study.

274-left sequence[1]	T	G	G	C	T	T	T	T	C	T	G	C	G	G	A	T
274C-left RVDs[2]		NN	NN	HD	NG	NG	NG	NG	HD	NG	NN	HD	NN	NN	NI	NG
274mC-left RVDs[3]		NN	NN	HD	NG	NG	NG	NG	HD	NG	NN	N*	NN	NN	NI	NG

274-right sequence[1]	T	A	G	G	C	T	T	G	C	A	A	T	C	A	A	C	G	A	T
274C-right RVDs[2]		NI	NN	NN	HD	NG	NG	NN	HD	NI	NI	NG	HD	NI	NI	HD	NN	NI	NG
274mC-right RVDs[3]		NI	NN	NN	HD	NG	NG	NN	HD	NI	NI	NG	HD	NI	NI	N*	NN	NI	NG

31-left sequence[1]	T	A	T	A	A	C	G	T	G	T	T	G	C	G	T	T	A	T	C	C	T	T	T	T	C	G
31C-left RVDs[2]		NI	NG	NI	NI	HD	NN	NG	NN	NG	NG	NN	HD	NN	NG	NG	NI	NG	HD	HD	NG	NG	NG	NG	HD	NN
31mC-left RVDs[3]		NI	NG	NI	NI	N*	NN	NG	NN	NG	NG	NN	N*	NN	NG	NG	NI	NG	HD	HD	NG	NG	NG	NG	N*	NN

31-right sequence[1]	T	**C**	A	C	C	C	T	T	G	C	C	G	A	C	A	C	C	A	A	C	A	C	T	C	T
31C-right RVDs[2]		HD	NI	HD	HD	HD	NG	NG	NN	HD	HD	NN	NI	HD	NI	HD	HD	NI	NI	HD	NI	HD	NG	HD	NG
31mC-right RVDs[3]		HD	NI	HD	HD	HD	NG	NG	NN	HD	N*	NN	NI	HD	NI	HD	HD	NI	NI	HD	NI	HD	NG	HD	NG

[1]Nucleotide sequences of target regions of TALENs used in this study are shown in 5'-3' order. Highly methylated cytosines are shown in red.

[2]Repeat variable diresidues (RVD) in each TALENs containing HD modules are shown. The first RVD for the 5' T is provided from a vector (Cermak et al, 2011).

[3]Repeat variable diresidues (RVD) in each TALENs containing N* modules are shown. N* modules are placed at positions corresponding to highly methylated cytosines (Figure 1**A** and **B**).

Supplementary Table S2. Off-target analysis of TALENs used in this study.

TALEN	chromosome	TAL 1 Score	TAL 2 Score	composit score	TAL 1 Start	TAL 2 Start	Spacer Length	TAL 1 Target	TAL 2 Target
274C	chr02	6.5	6.1	12.5	19220690	19220737	15	T GGCTTTTCTGCGGAT	T AGGCTTGCAATCAACGAT
	chr04	12.5	12.9	25.4	20079533	20079580	15	T GGCTTCTCTGCTGAC	T AGGCTTGCAGTCAATGAT
	chr09	19.2	7.6	26.8	19128213	19128152	29	T GGCTCTTTAACACCA	T AAACTTCCAATCAACGAT
	chr08	16.1	10.8	26.9	11880682	11880630	20	T GTTATTTCTCCACAT	T AGACATGAAATCAACAAT
	chr02	13.0	15.2	28.2	21577046	21577095	17	T AACTATTGTACAAAT	T AAGCTTCAAATCATCTAT
	chr11	15.0	13.5	28.6	19674798	19674847	17	T AACTATTATATAAAT	T AGGCTACAAATCAACTAT
	chr04	15.3	13.6	28.8	34036201	34036254	21	T AAATTTACTGCGTCT	T AAGCTTGCAAACAATGCT
	chr01	10.9	18.0	28.9	329932	329884	16	T GGCTTATCTGCAACT	T AACCATGCAACCAAGCAA
	chr03	16.5	12.9	29.4	30479788	30479841	21	T AGCTTTCAGAAGGAT	T AAACTTGCAAGCAAACAT
	chr08	15.4	13.9	29.4	18173198	18173149	17	T AACTCTCATTCAGAT	T AGACATACAATTAACAAA
274mC	chr02	7.0	6.6	13.5	19220690	19220737	15	T GGCTTTTCTGCGGAT	T AGGCTTGCAATCAACGAT
	chr04	13.0	10.5	23.5	20079533	20079580	15	T GGCTTCTCTGCTGAC	T AGGCTTGCAGTCAATGAT
	chr11	12.7	14.0	26.7	19674798	19674847	17	T AACTATTATATAAAT	T AGGCTACAAATCAACTAT
	chr04	15.8	11.3	27.0	34036201	34036254	21	T AAATTTACTGCGTCT	T AAGCTTGCAAACAATGCT
	chr09	19.7	8.1	27.8	19128213	19128152	29	T GGCTCTTTAACACCA	T AAACTTCCAATCAACGAT
	chr08	11.1	16.7	27.8	608843	608895	20	T GACTTTTCTATACCT	T ACCCTTGCCATCAATTGT
	chr08	16.6	11.3	27.9	11880682	11880630	20	T GTTATTTCTCCACAT	T AGACATGAAATCAACAAT
	chr03	10.2	18.0	28.2	32426303	32426362	27	T AACTTCTCTATGAAT	T ACTCTTACCATCCAAGAA
	chr03	9.8	18.4	28.2	14654648	14654702	22	T AACTTTTCTTTGGAT	T AAGCATGAAGTGAACAAT
	chr01	11.4	16.9	28.3	329932	329884	16	T GGCTTATCTGCAACT	T AACCATGCAACCAAGCAA
31C	chr02	8.67	5.65	14.3	19220447	19220514	19	T ATAACGTGTTGCGTTATCCTTTTCG	T CACCCTTGCCGACACCAACACTCT
31mC	chr02	10.17	6.16	16.3	19220447	19220514	19	T ATAACGTGTTGCGTTATCCTTTTCG	T CACCCTTGCCGACACCAACACTCT

Off-target sites were surveyed with Paired Target Finder at TAL Effector Nucleotide Targeter 2.0 (https://tale-nt.cac.cornell.edu/) in which N* module is considered to have potential to bind to C and T (Moscou and Bogdanove, 2009; Doyle et al., 2012). TALENs for target 274 give many potential off-target sites with low probabilities and only top 10 sites are shown for each 274C-TALENs and 274mC-TALENs. TALENs for target 31 give only the true target under a default condition (score cutoff = 3). Because *Fok*I portions that work specifically in a dimer were used for TALENs (Miller et al., 2007; Zhang et al., 2013), only heterodimer targets were considered.

References not shown in the main text

Doyle, E. L., Booher, N. J., Standage, D. S., Voytas, D. F., Brendel, V. P., VanDyk, J. K., et al. (2012). TAL effector-nucleotide targeter (TALE-NT) 2.0: tools for TAL effector design and target prediction. *Nucleic Acids Res.* 40, W117-W122. doi: 10.1093/nar/gks608

Miller, J. C., Holmes, M. C., Wang, J., Guschin, D. Y., Lee, Y.-L., Rupniewsk,i I., et al. (2007). An improved zinc-finger nuclease architecture for highly specific genome editing. *Nat. Biotech.* 25, 778-785. doi: 10.1038/nbt1319

Supplementary Table S3. Frequency of TALENs-induced mutations in *ACS1*.

target	TALENs	number of independent calli analyzed	number of calli carrying mutation	mutation frequency (%)	p *
274	274C-TALENs	138	2	1.45	0.019
	274mC-TALENs	99	8	8.08	
31	31C-TALENs	104	1	0.96	0.466
	31mC-TALENs	119	0	0.00	

*Fisher's exact test

Supplementary Table S4. Summary of mutation frequencies and CG methylation rates around target 274 in calli carrying a control TALENs and 274mC-TALENs.

		CG methylation rate (%)[1]					
	position (bp)[2]	c1[3]	c2[3]	1[3]	6[3]	7[3]	97[3]
	−12	83	79.2	100	91.3	57.5	69.7
	11	100	95.8	97.9	71.7	89.7	80
number of total clones analyzed[4]		47	48	47	46	44	46
number of clones analyzed[5]	−12	47	48	47	46	42	33
	11	47	48	47	46	41	40
mutation (%)[6]		0	0	0	19.6	38.6	100
number of mutated sequences[7]		0	0	0	2	6	20

[1]Methylation frequencies of cytosines in the CG context at indicated positions.

[2]Position of cytosine in the CG context relative to center of two TALEN monomers targeted to an *ACS1* region.

[3]Callus clones analyzed (c1 and c2, control TALENs; 1, 6, 7, and 97, 274mC-TALENs).

[4]Number of independent PCR clones analyzed for detection of mutations.

[5]Number of independent PCR clones analyzed for methylation rates of cytosines at positions −12 and +11 in bisulfite sequencing.

[6]Mutation rate at target 274.

[7]Number of differently mutated sequences detected in each callus.

Supplementary Table S5. Effect of genome editing on methylation of cytosine at +11 position in target 274.

sample name	number of clones analyzed	number of methylated cytosine	number of unmethylated cytosines	methylation rate	samples showing significant differences in Ryan's test[1]
c1	47	47	0	1.00	6, 97
c2	48	46	2	0.96	6
1	47	46	1	0.98	6, 97
6	46	33	13	0.72	**c1, c2, 1**
7	41	37	4	0.90	none
97	40	32	8	0.80	c1, 1

[1] Samples with significant differences in methylation rates are shown. Sample No. 6 shows significantly different methylation levels to those in two controls (c1 and c2) and a callus (No. 1) without detectable levels of genome editing (indicated in red letters).

Supplementary Table S6. Primers used in this study.

primer	sequence	used for
TALEN-ACS1-1	GTGGTTCAAAGGTGGCAAAACAAAC	CAPS analysis of mutations in *ACS1*
TALEN-ACS1-2	AGCTTAGAATTGCCTGTGCGGATGC	CAPS analysis of mutations in *ACS1*
FokI-F	TACGGTGTGATCGTGGATACTAA	RT-PCR
FokI-R	TGTGCCGGCTTTAATCATTTCTC	RT-PCR
NPTII-F	TGGAGAGGCTATTCGGCTATGACTGGGCACAA	RT-PCR
NPTII-R	TCAGAAGAACTCGTCAAGAAGGCGATAGAAGG	RT-PCR
ACTIN-F	TAACAGATAGGCCGGTTGAAAAC	RT-PCR
ACTIN-R	CATGAAGTGCGACGTGGATATTA	RT-PCR
Me-ACS1-F11	AATGTGTGTYATTGGATGTTTGTGGAAAAT	bisulfite analysis (primary PCR)
Me-ACS1-F22	GAAGAGTGTTGGTGTYGGYAAGGGTGATG	bisulfite analysis (nested PCR)
Me-ACS1-R11	CCARACCARCATCCACTATATCTTTCA	bisulfite analysis (nested PCR)
Me-ACS1-R22	CATACCTACARCRACTCCTTRTTTCTCACT	bisulfite analysis (primary PCR)

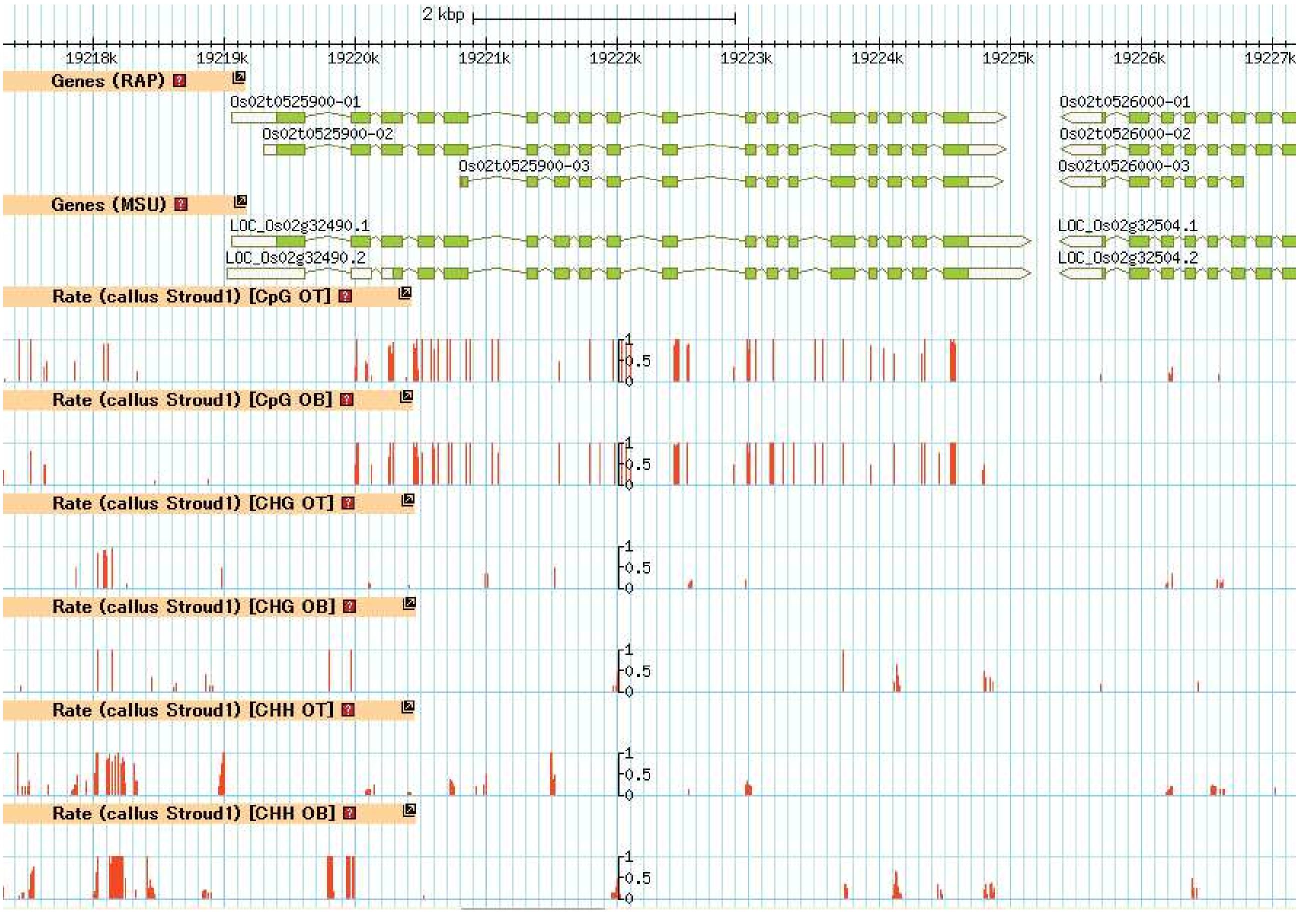

Supplementary Figure S1. Methylation state of *ACS1* gene region.

Methylation rates (red bars) of cytosines in CpG, CHG, and CHH contexts in *ACS1* gene region are separately shown. Exon-intron structures annotated in RAP and MSU databases are shown. OT, top strand; OB, bottom strand. BS-seq data of rice callus were taken from GSM1039499 (Stroud et al, 2013).

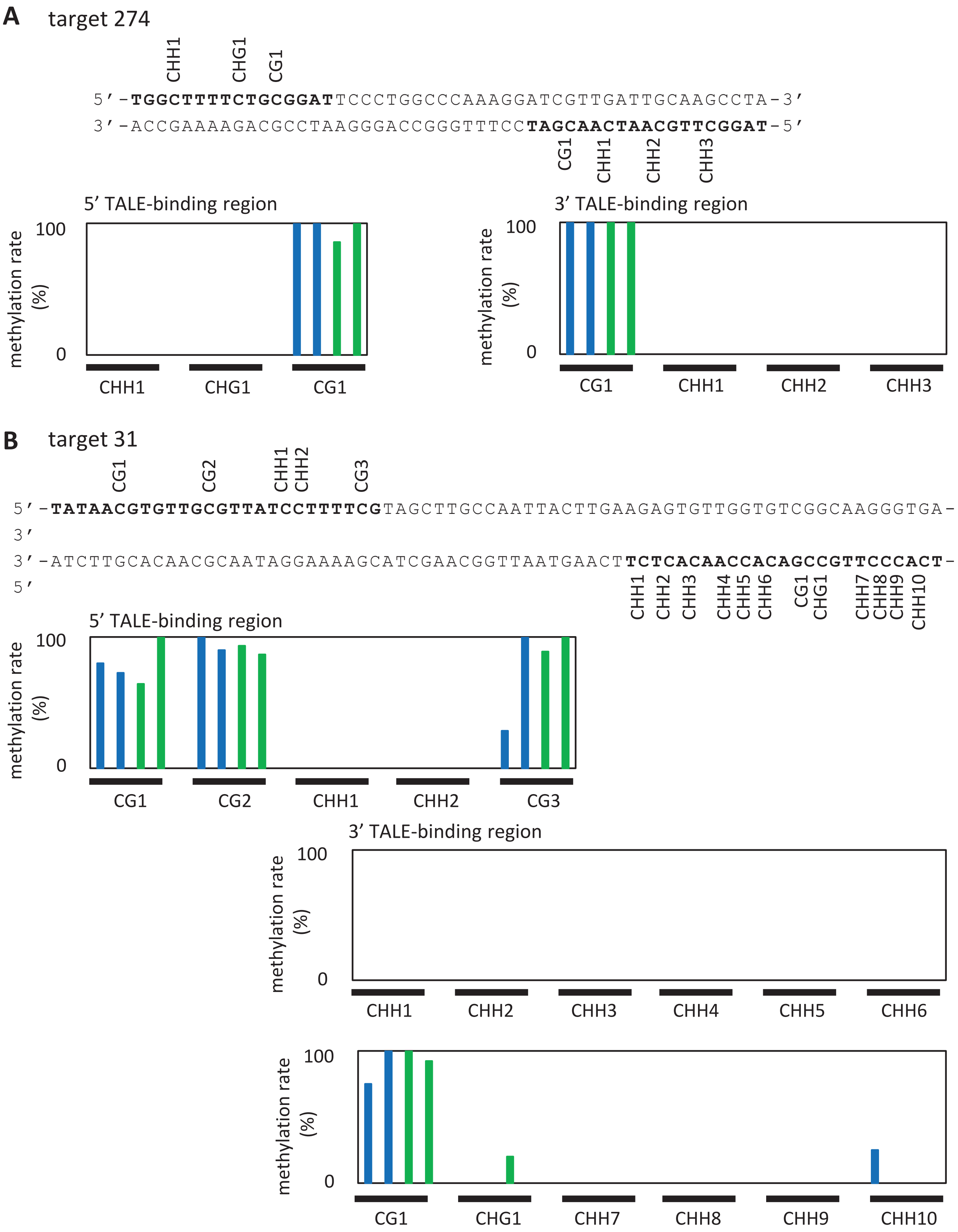

Supplementary Figure S2. Methylation rates of individual cytosines in the target regions in *ACS1*. Methylation rates of cytosines in the target regions of TALENs used in this study were extracted from four independent BS-seq data of wild-type rice calli (blue bars, Stroud et al 2013; green bars, BS-seq that was done in this study with two independent wild-type Nipponbare callus samples (4 weeks after callus induction) as described previously (Numa et al 2015) . Positions of cytosines in the TALE-binding regions are shown with the context of each cytosine (CG, CHG, or CHH). (**A**) target 274; (**B**) target 31.

Reference not shown in the main text

Numa, H., Yamaguchi, K., Shigenobu, S., Habu, Y. (2015) Gene body CG and CHG methylation and suppression of centromeric CHH methylation are mediated by DECREASE IN DNA METHYLATION1 in rice. *Mol. Plant* 8(10):1560-1562. doi: 10.1016/j.molp.2015.08.002

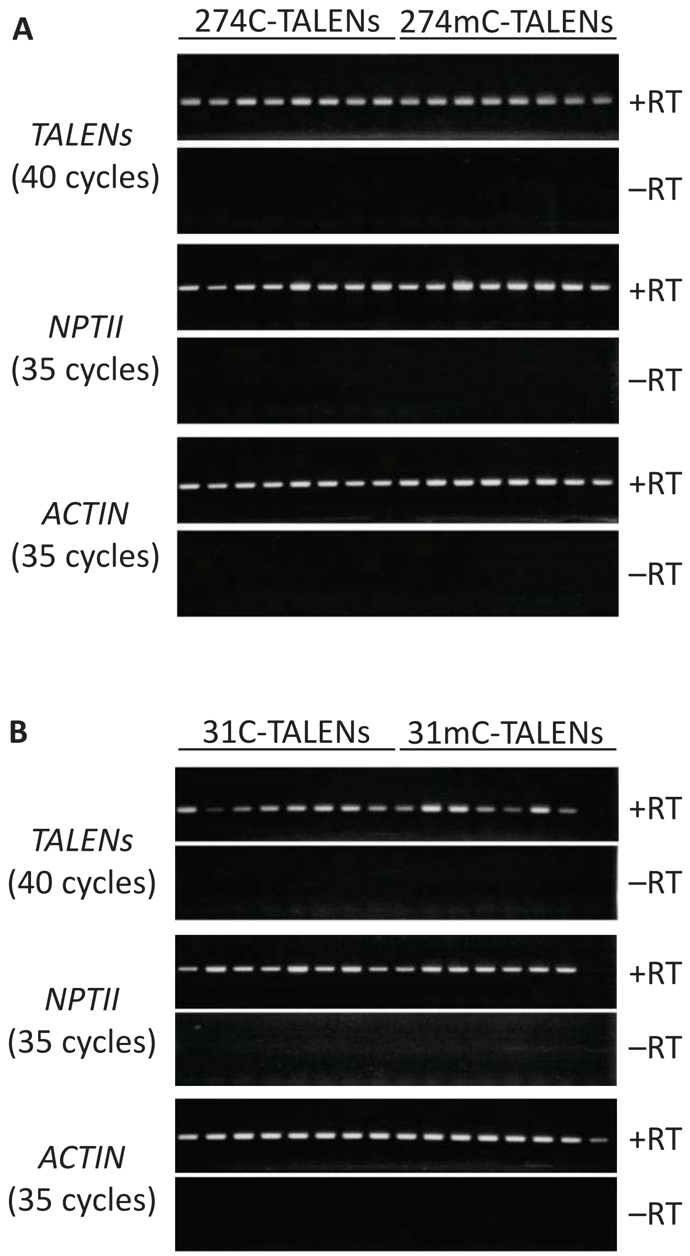

Supplementary Figure S3. RT-PCR of introduced TALENs constructs.
RT-PCR was performed for *TALENs* (upper), *NPTII* (middle), and endogenous *ACTIN* (bottom) genes. (**A**) calli transformed with TALENs constructs for target 274; (**B**) calli transformed with TALENs constructs for target 31.

A

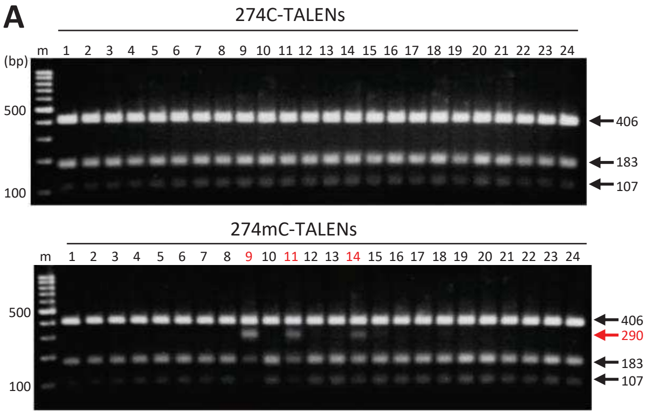

B

```
                          left binding site                  right binding site            del     n
wild-type    CTCAGGTTGTATTTGCTGGCTTTTCTGCGGATTCCCTGGCCCAAAGGATCGTTGATTGCAAGCCTAAGCTTGTGATCACT
mutant  #1   CTCAGGTTGTATTTGCTGGCTTTTCTGCGGATTCCCTGGC--AAAGGATCGTTGATTGCAAGCCTAAGCTTGTGATCACT   -2   ( ×2)
mutant  #2   CTCAGGTTGTATTTGCTGGCTTTTCTGCGGATTCCCTG---CAAAGGATCGTTGATTGCAAGCCTAAGCTTGTGATCACT   -3   ( ×2)
mutant  #3   CTCAGGTTGTATTTGCTGGCTTTTCTGCGGATTCCCT----CAAAGGATCGTTGATTGCAAGCCTAAGCTTGTGATCACT   -4   ( ×1)
mutant  #4   CTCAGGTTGTATTTGCTGGCTTTTCTGCGGATTCCCTG----AAAGGATCGTTGATTGCAAGCCTAAGCTTGTGATCACT   -4   ( ×3)
mutant  #5   CTCAGGTTGTATTTGCTGGCTTTTCTGCGGATTCCC-------AAGGATCGTTGATTGCAAGCCTAAGCTTGTGATCACT   -7   ( ×1)
mutant  #6   CTCAGGTTGTATTTGCTGGCTTTTCTGCGGA---------CCAAAGGATCGTTGATTGCAAGCCTAAGCTTGTGATCACT   -9   ( ×2)
mutant  #7   CTCAGGTTGTATTTGCTGGCTTTTCTGCGGATTC----------AGGATCGTTGATTGCAAGCCTAAGCTTGTGATCACT  -10   ( ×4)
mutant  #8   CTCAGGTTGTATTTGCTGGCTTTTCTGC------------CCAAAGGATCGTTGATTGCAAGCCTAAGCTTGTGATCACT  -12   ( ×1)
mutant  #9   CTCAGGTTGTATTTGCTGGCTTTTCTGCGGAT-------------GGATCGTTGATTGCAAGCCTAAGCTTGTGATCACT  -13   ( ×1)
mutant #10   CTCAGGTTGTATTTGCTGGCTTTTC-------------------------GTTGATTGCAAGCCTAAGCTTGTGATCACT  -25   (×12)
mutant #11   CTCAGGTTGTATTTGCTGGCTT-----------------------------TTGATTGCAAGCCTAAGCTTGTGATCACT  -29   ( ×1)
mutant #12   CTCAGGTTGTATTTGCTGGCTTTTC-------------------------------TGCAAGCCTAAGCTTGTGATCACT  -31   ( ×1)
mutant #13   CTCAGGTTGTATT------------------------------------CGTTGATTGCAAGCCTAAGCTTGTGATCACT  -36   ( ×1)
mutant #14   CTCAGGTTGTA-------------------------------------TCGTTGATTGCAAGCCTAAGCTTGTGATCACT  -37   ( ×1)
mutant #15   C-----------------------------------------AAAGGATCGTTGATTGCAAGCCTAAGCTTGTGATCACT  -41   ( ×1)
mutant #16   CTCAGGTTG---------------------------------------------ATTGCAAGCCTAAGCTTGTGATCACT  -45   ( ×1)
mutant #17   CTCAG---------------------------------------------GTTGATTGCAAGCCTAAGCTTGTGATCACT  -45   ( ×9)
```

C

```
                                                                                           in      n
 wild-type   CTCAGGTTGTATTTGCTGGCTTTTCTGCGGATTCCCTG GCCCAAAGGATCGTTGATTGCAAGCCTAAGCTTGTGATCACT
mutant #18   CTCAGGTTGTATTTGCTGGCTTTTCTGCGGATTCCCTGTGCCCAAAGGATCGTTGATTGCAAGCCTAAGCTTGTGATCACT   +1   ( ×4)
mutant #19   CTCAGGTTGTATTTGCTGGCTTTTCTGCGGATTCCCTGGGCCCAAAGGATCGTTGATTGCAAGCCTAAGCTTGTGATCACT   +1   ( ×1)
```

D

```
 wild-type   AGCTCTCGTGGTTATCCTGGTTAATCCTTATGATTTAATGCATACTGCAAAAATTTCTCAGGTTGTATTTGCTGGCTTTT
mutant #20   AGCTCTCGTGGTTATCCTGGTTAATCCTTATGATTTAATGCATACTGCAAAAATTTCTCAGGTTGTATTTGCTGGCTTTT
mutant #21   AGCTCTCGTGGTTATCCTGGTTAATCCTTATGATTTAATGCATACTGCAAAAATTTCTCAGGTTGTATTTGCTGGCTTTT

 wild-type   CTGCGGATTCCCTGGC               CCAAAGGATCGTTGATTGCAAGCCTAAGCTTGTGATCACTTGCAATGCA
mutant #20   CTGCGGATTCCCTGGATATAGTGGATGCTGGTCTGGCTGAAAGTGATTGCAAGCCTAAGCTTGTGATCACTTGCAATGCA
mutant #21   CTGC        AAAAATATCCTGGTTAATCCTTAC   GATCGTTGATTGCAAGCCTAAGCTTGTGATCACTTGCAATGCA

                                                                            in/del    n
 wild-type   GTCAAAAGAGGGGTGAAGCCCATCCTTCTGAAAGATATAGTGGATGCTGGTCTGGCTGAAAGTGAGA
mutant #20   GTCAAAAGAGGGGTGAAGCCCATCCTTCTGAAAGATATAGTGGATGCTGGTCTGGCTGAAAGTGAGA  +28/-13  (×1)
mutant #21   GTCAAAAGAGGGGTGAAGCCCATCCTTCTGAAAGATATAGTGGATGCTGGTCTGGCTGAAAGTGAGA  +24/-18  (×1)
```

Supplementary Figure S4. Mutations induced by 274mC-TALENs.

(**A**) CAPS analysis of target 274 region in calli carrying 274C- or 274mC-TALENs. Changes in nucleotide sequences at the target sequence are expected to produce DNA fragments around 290 bp. Upper, 274C-TALENs containing cytosine-binding modules; lower, 274mC-TALENs containing methylcytosine-binding modules; m, a 100 bp-size marker. Results of representative 24 colonies for each TALENs are shown. Numbers of calli showing sequence alterations at the target site are indicated in red. (**B**) Sequences of deletions induced by 274mC-TALENs. Numbers of deleted nucleotides (del) and numbers of PCR clones carrying corresponding sequences (n) are indicated to the right. Left and right binding sites of 274mC-TALENs are shown in red. A *Hae*III site (GGCC) located at the center of left- and right-binding sites was used for the CAPS analysis. (**C**) Sequences of insertions induced by 274mC-TALENs. Numbers of inserted nucleotides (in) are indicated to the right. Inserted nucleotides are indicated in blue. (**D**) Sequence rearrangements induced by 274mC-TALENs. Nucleotides inserted into the target site are indicated in brown or green for each clone. Positions of sequences that are homologous to the inserted sequences are indicated in same colors. Sizes of insertions/deletions (in/del) are shown in the right.

A 31C-TALENs

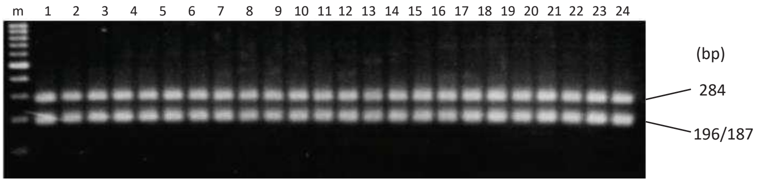

B 31mC-TALENs

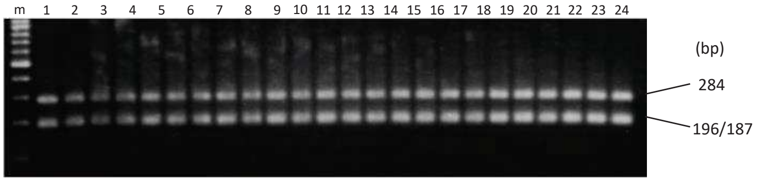

Supplementary Figure S5. CAPS analysis of target 31 region.
Changes in nucleotide sequences at the target sequence are expected to produce DNA fragments around 470 bp. (**A)** 31C-TALENs containing cytosine-binding modules; (**B)** 31mC-TALENs containing methylcytosine-binding modules; m, a 100 bp-size marker. Results of representative 24 colonies for each TALENs are shown.

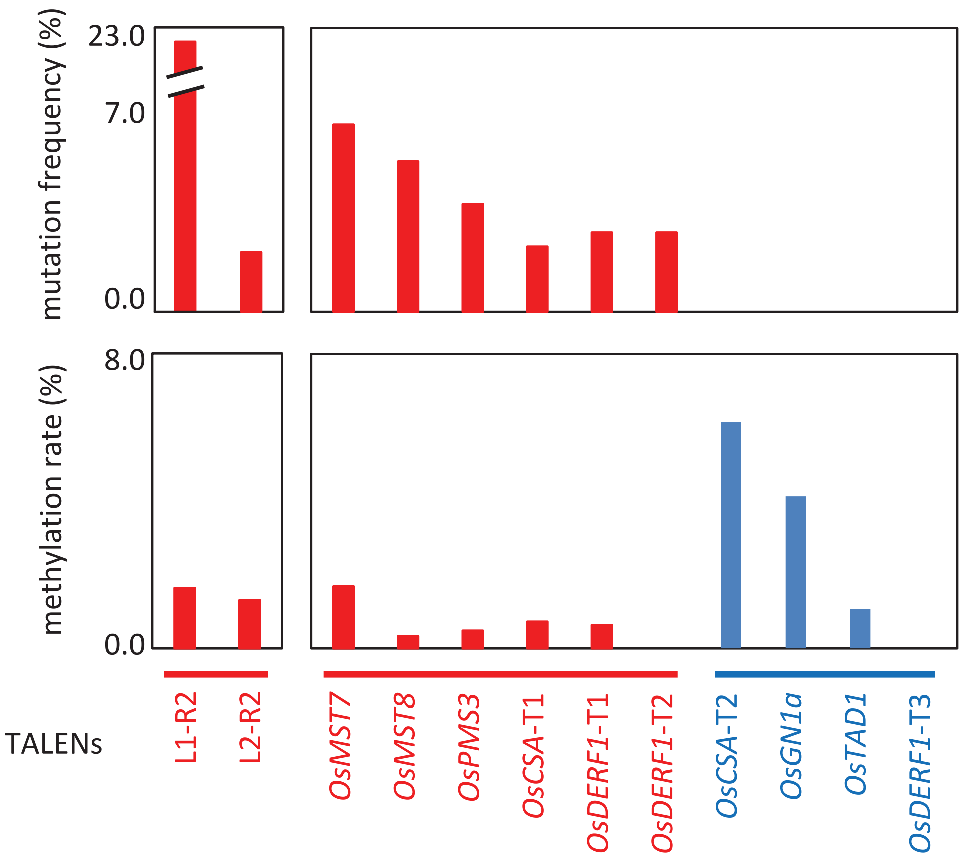

Supplementary Figure S6. Inverse correlation between mutation frequencies in TALEN-mediated genome editing and methylation rates of cytosines in the target regions in rice. (upper) Mutation frequencies of TALEN-mediated genome editing taken from published studies. L1-R2 and L2-R2, Nishizawa-Yokoi et al (2016); others, Zhang et al (2016). (lower) Methylation rates of cytosines in the target regions. Methylation rates of the target regions in wild-type rice calli were calculated by combining four independent BS-seq data: two from Stroud et al (2013) and two obtained in this study. Names of TALENs are shown at the bottom with their target genes/regions and nucleotide sequences of the target regions are shown in Supplementary Figure S7. Those shown in red are TALENs with detectable genome editing activities. L1-R2 has a higher genome editing activity, indicating that there would be factors other than cytosine methylation that affect efficiency of TALENs.

A *OsMST7*

5′-**TGCTTGGGCGCGCCATCGCC**GGCGCCGGCGTTCTCCATTGCCAGGGAAACGAGA-3′
3′-ACGAACCCGCGCGGTAGCGGCCGCGGCCGCAAGAGG**TAACGGTCCCTTTGCTCT**-5′

OsMST8

5′-**TCGGAATTCCCATCAT**GGCCGGCGGCGCCATGACCGACACCGATGGCGCTCA-3′
3′-AGCCTTAAGGGTAGTACCGGCCGCCGCGGTACTGG**CTGTGGCTACCGCGAGT**-5′

OsTAD1

5′-**TCCTCCTCCGCGCCCACGCC**GGCCTCGCGGACGGTCTACAGCGACCGCTTCATCCCCA-3′
3′-AGGAGGAGGCGCGGGTGCGGCCGGAGCGCCTGCCAG**ATGTCGCTGGCGAAGTAGGGGT**-5′

OsGN1a

5′-**TTGAGCATGAGGAGCACTGCC**ATCCTGACCTGCTCTTGCTTGATTATCAATCAATCA-3′
3′-AACTCGTACTCCTCGTGACGGTAGGACTGGACGAGAACG**AAGTAATAGTTAGTTAGT**-5′

OsDERF1-T1

5′-**TCTTCCGTGCACTATAAGT**ATAATCTCTCACTCTCTCCTCTTTGATCATCATCAGCTCA-3′
3′-ACAAGGCACGTGATATTCATATTAGAGAGTGAGAGAG**GAGAAACTAGTAGTAGTCGAGT**-5′

OsDERF1-T2

5′-**TGCGTACATAGGAGAG**TGTGTTTCTTGCCTATATTAGTGATTAGTTGCGA-3′
3′-ACGCATGTATCCTCTCACACAAAGAACGGATA**TAATCACTAATCAACGCT**-5′

OsDERF1-T3

5′-**TTGGAATTAGCGGCGGCGGCGA**GGGCGGGGTGGTGTCTCCGGCGCAGCCGTCGTCGCCGGAGCGGCGGTA-3′
3′-AACCTTAATCGCCGCCGCCGCTCCCGCCCCACCACAGAGGCCGC**GTCGGCAGCAGCGGCCTCGCCGCCAT**-5′

OsCSA-T1

5′-**TGGATGGAGACCGACCT**CGCGGTGGCCTATATAAGAGAAGAGAAGAGGA-3′
3′-ACCTACCTCTGGCTGGAGCGCCACCGGATATA**TTCTCTTCTCTTCTCCT**-5′

OsCSA-T2

5′-**TCCACAGCAAGCTTCAAGAAATGCT**GGAATGGATGGATGGATGGAGACCGACCTCGCGGTGGCCTATATA-3′
3′-AGGTGTCGTTCGAAGTTCTTTACGACCTTACCTACCTACCTACCTCTGG**CTGGAGCGCCACCGGATATAT**-5′

OsPMS3

5′-**TTTAGGCATGTGTCTTA**GGGTTTTTAATGGAACTTCATGGGTTGTTAGA-3′
3′-AAATCCGTACACAGAATCCCAAAAATTACC**TTGAAGTACCCAACAATCT**-5′

B L1/R2

5′-**TCCTTATAAGCACATAT**GGCATTGTAATATATATGTTTGAGTTTTAGCGACA-3′
3′-AGGAATATTCGTGTATACCGTAACATTATATA**TACAAACTCAAAATCGCTGT**-5′

L2/R2

5′-**TGCTCCTTAAGTCCTTATAAGCACATAT**GGCATTGTAATATATATGTTTGAGTTTTAGCGACA-3′
3′-ACGAGGAATTCAGGAATATTCGTGTATACCGTAACATTATATA**TACAAACTCAAAATCGCTGT**-5′

Supplementary Figure S7. Nucleotide sequences of target regions of TALEN-mediated genome editing.
(**A**) Zhang et al (2016) and (**B**) Nishizawa-Yokoi et al (2016). Nucleotides in left and right TALEN-binding regions are shown in bold letters.
